# Hemiballismus as the Sole Manifestation of Acute Thalamic Hemorrhagic Stroke: A Case Report

**DOI:** 10.7759/cureus.93719

**Published:** 2025-10-02

**Authors:** Yoseph M Habte, Binyam M Habte, Esimael M Abdu, Abdulkerim A Temam, Amira A Mohammed

**Affiliations:** 1 Department of Medicine, Ethio Tebib Hospital, Addis Ababa, ETH; 2 Department of Medicine, University of Gondar, Gondar, ETH; 3 Department of Surgery, Teklehaimanot General Hospital, Addis Ababa, ETH; 4 Department of Emergency Medicine, Ethio Tebib Hospital, Addis Ababa, ETH

**Keywords:** case report, hemiballismus, hemorrhagic stroke, post-stroke movement disorder, thalamic hemorrhage

## Abstract

Post-stroke movement disorders are uncommon, with hyperkinetic types occurring in less than 1% of cases. Hemiballism-hemichorea is the most frequent post-stroke hyperkinetic movement disorder and is typically associated with lesions in subcortical structures. We report a case of a 68-year-old man with hypertension, diabetes, and prior ischemic stroke who developed right-sided hemiballismus within days of a left thalamic hemorrhagic stroke. Brain MRI revealed a hemorrhagic lesion in the left thalamus alongside chronic small vessel ischemic changes and lacunar infarcts. The patient exhibited continuous, high-amplitude involuntary movements that significantly impaired function. Symptomatic treatment with haloperidol, sodium valproate, and clonazepam led to partial improvement, though complete resolution was not achieved during the hospitalization. This case highlights the clinical complexity of post-stroke hemiballismus, the broad neuroanatomical substrates beyond the subthalamic nucleus, and challenges in management, particularly in patients with multiple vascular comorbidities. Given the variability in onset and recovery, close neurological follow-up and individualized therapy are essential. Further research is warranted to better understand the pathophysiology and optimize treatment strategies for post-stroke hyperkinetic movement disorders.

## Introduction

Post-stroke movement disorders are relatively uncommon in the adult population. Although comprehensive data are limited, it is generally estimated that less than 1% of stroke patients develop hyperkinetic movement disorders, either acutely or with delayed onset [1]. These disorders encompass a broad spectrum of clinical presentations, including both hypokinetic and hyperkinetic syndromes. Hyperkinetic manifestations, in particular, often occur in varying combinations and are typically categorized into three phenotypes: choreiform dyskinesias (such as ballism, chorea, and athetosis), dystonia, and non-choreo-dystonic dyskinesias (including tremor, asterixis, and myoclonus) [2].

Hemiballism-hemichorea is recognized as the most frequently observed movement disorder following stroke, accounting for approximately 40% of cases in one case series [1,3]. Evidence suggests that hemorrhagic strokes are more commonly associated with post-stroke movement disorders compared with ischemic events [4]. Hemiballism is clinically defined by forceful, irregular, poorly coordinated, high-amplitude movements affecting the limbs on one side of the body, and is generally considered a more severe manifestation within the choreiform spectrum [5]. Disorders such as chorea and hemiballism typically emerge within hours to several days after the cerebrovascular insult, although considerable variability in onset timing exists across different movement disorder subtypes. This heterogeneity may have implications for both the window of partial motor recovery and the maladaptive reorganization of neural pathways [1,5]. Notably, approximately 90% of acute post-stroke movement disorders tend to show spontaneous resolution within six months [1,3].

We present a case of a 68-year-old man with a history of hypertension, diabetes mellitus, and a prior ischemic stroke, who developed right-sided hemiballismus secondary to a left thalamic hemorrhagic stroke. This case highlights the clinical presentation, imaging findings, and therapeutic challenges of managing post-stroke hemiballismus in a patient with multiple comorbidities.

## Case presentation

A 68-year-old male with a 10-year history of hypertension and type 2 diabetes mellitus, managed with metformin 500 mg orally twice daily, glibenclamide 5 mg orally daily, and a combination of amlodipine/valsartan 5/160 mg orally daily, presented with a three-day history of progressive, involuntary, high-amplitude, purposeless movements involving the right upper and lower extremities. The movements were continuous and disabling, resulting in a fall without loss of consciousness. There was no associated headache, fever, or altered mentation.

His past medical history included left-sided hemiparesis secondary to ischemic stroke four months earlier, which left him with residual slurring of speech. He had been maintained on aspirin 81 mg orally daily and atorvastatin 40 mg orally daily for secondary stroke prevention. 

On examination, he was alert and oriented, with a Glasgow Coma Scale score of 15/15. Vital signs were as follows: blood pressure 150/90 mmHg, pulse 82 beats per minute, respiratory rate 20 breaths per minute, and oxygen saturation fluctuating between 90%-94% on room air. There was no clinical evidence of respiratory compromise or underlying pulmonary pathology, and the desaturations were transient. Cardiovascular and respiratory system examinations were unremarkable. Neurological assessment revealed continuous, irregular, high-amplitude involuntary movements affecting the right upper and lower limbs, consistent with hemiballismus. Muscle strength was preserved (5/5) bilaterally, and there was no deterioration of mentation.

Laboratory investigations showed leukocytosis (WBC: 12.3 x 10³/μL), while the coagulation profile, electrolytes, renal function tests, and liver function tests were within their normal reference ranges. Additionally, HbA1c was measured at 5.3%, indicating good glycemic control (Table 1).

**Table 1:** Laboratory investigations with corresponding results and reference values

Laboratory parameter	Result	Normal value
Complete blood count		
WBC	12.3 x 10^3^/µL	4.0-11.0 x 10^3^/µL
Hemoglobin	13.5 g/dL	13.5-17.5 g/dL
Platelet	253 x 10^3^/µL	150-450 x 10^3^/µL
Lymphocyte percentage	15.3%	15%-50%
Neutrophil percentage	74.9%	45%-80%
Metabolic panel		
Creatinine	1.10 mg/dL	0.67-1.17 mg/dL
Urea	40.3 mg/dL	17-43 mg/dL
Na^+^	136 mmol/L	136-145 mmol/L
K^+^	3.60 mmol/L	3.5-5.1 mmol/L
Albumin	3.86 g/dL	3.5-5.2 g/dL
Total protein	6.89 g/dL	6.6-8.3 g/dL
Aspartate transaminase	48.6 U/L	2-50 U/L
Alanine transaminase	30.3 U/L	1-50 U/L
Coagulation profile		
Prothrombin time	14.3 seconds	10.7-14.3 seconds
International normalized ratio	1.16	0.8-1.2
Activated partial thromboplastin time	32.1	21-35 seconds
Peripheral blood smear	Normal morphology	
HbA1c	5.3%	4.0%-6.5%

**Figure 1:**
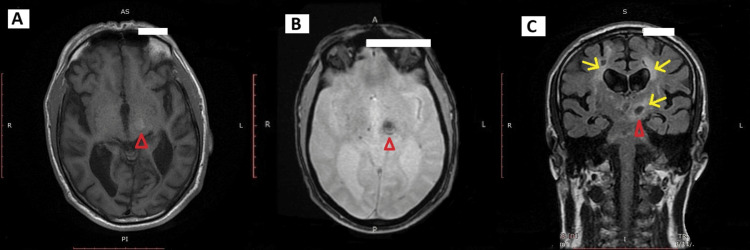
MRI of the brain demonstrating thalamic hemorrhage and chronic small vessel ischemic changes Multiplanar brain MRI demonstrating a 1.3 cm subacute hemorrhagic lesion centered in the left thalamus and superior cerebral crus (red arrowhead), appearing: (A) Hyperintense on axial T1-weighted imaging (B) Hypointense on axial gradient-recalled echo imaging (C) Hypointense on coronal T2/FLAIR imaging Also noted are moderate-to-severe chronic small vessel ischemic changes, with periventricular and deep white matter hyperintensities (yellow arrows), along with cerebral atrophy.

MRI of the brain revealed a 1.3 cm subacute hemorrhagic lesion centered in the left thalamus and the superior portion of the left cerebral crus. The lesion was hyperintense on T1 sequences and hypointense on T2/FLAIR and gradient-recalled echo sequences, suggestive of blood products. Additional findings included moderate-to-severe chronic small vessel ischemic changes in the periventricular and deep white matter, associated with cerebral atrophy, and multiple lacunar infarcts in the bilateral centrum semiovale, basal ganglia, and pons (Figure 1).

He was admitted to the high-dependency unit with a working diagnosis of right-sided hemiballismus secondary to a left thalamic hemorrhagic stroke, superimposed on a background of chronic ischemic cerebrovascular disease. 

Symptomatic treatment for hemiballismus was initiated with haloperidol 2.5 mg orally daily and later escalated to 2.5 mg orally twice daily due to persistent symptoms. Sodium valproate 200 mg orally twice daily and clonazepam 0.25 mg orally at night were subsequently added following neurology consultation, resulting in partial symptom improvement.

During hospitalization, the patient's hemiballistic movements gradually decreased in intensity but did not fully resolve. He remained clinically stable, tolerated oral feeding without aspiration, and exhibited no new neurological deficits. He was discharged on the eighth day of admission in an improving condition, with a treatment regimen that included continued use of haloperidol, sodium valproate, and clonazepam. He was advised to follow up closely with neurology, internal medicine, and urology clinics.

At a two-week outpatient follow-up, the patient continued to experience right-sided hemiballismus without further improvement. Based on clinical reassessment, his clonazepam dose was increased to 0.5 mg nightly, sodium valproate was reduced to 200 mg daily, and haloperidol was maintained at 2.5 mg twice daily.

## Discussion

Post-stroke movement disorders, although uncommon, can present with a variety of clinical phenotypes, with hemiballismus-hemichorea being the most frequently observed hyperkinetic syndrome [1,2]. This case of a 68-year-old man with right-sided hemiballismus secondary to a left thalamic hemorrhagic stroke highlights the complex neuroanatomical and pathophysiological underpinnings of post-stroke hyperkinetic movements, as well as the challenges encountered in clinical management.

The classical understanding links hemiballismus to lesions of the subthalamic nucleus; however, accumulating evidence reveals that lesions in other subcortical areas, such as the thalamus, basal ganglia, and their associated neural networks, can also precipitate this movement disorder [1,3]. In our patient, MRI demonstrated a hemorrhagic lesion centered in the left thalamus, accompanied by lacunar infarcts and microvascular changes, consistent with hypertensive small vessel disease. These findings support the concept that disruption within a broader cortico-basal ganglia-thalamo-cortical circuitry contributes to hemiballismus [6].

At the neurochemical level, abnormal hyperkinetic movements may result from altered inhibitory GABAergic transmission and dysregulated dopaminergic pathways within these networks [7]. Such neurotransmitter imbalances likely lead to impaired basal ganglia output synchronization, which manifests clinically as the high-amplitude, irregular, and purposeless movements characteristic of hemiballismus [5]. This pathophysiological complexity is exemplified by cases, including ours, where the lesion lies outside the subthalamic nucleus but produces similar clinical features, underscoring the role of a sophisticated, interconnected neural network in generating these symptoms [8].

The patient’s pre-existing vascular risk factors, hypertension, diabetes mellitus, and prior ischemic stroke likely contributed both to the initial hemorrhagic event and the cumulative small vessel disease burden, as evidenced by microbleeds and lacunar infarcts observed on imaging. These comorbidities not only increase the risk of stroke but may also influence lesion evolution and the clinical presentation of post-stroke movement disorders, complicating management and recovery [9].

Imaging remains crucial in confirming the diagnosis of stroke and identifying lesion location; however, its role in predicting clinical manifestations of hemiballismus is limited, as lesions in diverse brain regions can yield similar symptoms [10]. In our case, the lesion’s location in the left thalamus and adjacent structures aligns with the disruption of key motor circuits, providing an anatomical basis for the observed right-sided hemiballismus. The additional imaging findings of cerebral microbleeds and chronic small vessel ischemic changes further reflect an underlying diffuse vascular burden, which may contribute to the functional disinhibition of motor pathways.

Therapeutically, the management of post-stroke hemiballismus remains challenging due to the lack of standardized guidelines and the typically transient nature of symptoms. In our patient, symptomatic treatment with haloperidol, sodium valproate, and clonazepam led to partial improvement but not complete resolution of involuntary movements. Dopamine receptor antagonists such as haloperidol have historically demonstrated efficacy in resolving hemiballismus, with studies reporting symptom resolution in over half of patients within two weeks [5]. Benzodiazepines and antiseizure medications, including clonazepam and valproate, offer alternative or adjunctive benefits by modulating GABAergic neurotransmission [8].

For refractory or severe cases, non-pharmacological options such as deep brain stimulation (DBS) targeting the internal globus pallidus, ventral intermediate nucleus of the thalamus, or subthalamic nucleus have shown promising results, albeit with potential complications [11]. While DBS was not indicated in this patient, it remains a viable consideration for persistent disabling symptoms.

Regarding prognosis, approximately 90% of post-stroke movement disorders resolve within six months, but symptom persistence beyond the acute phase necessitates ongoing clinical monitoring and tailored therapy [1,3]. In this patient, the partial symptom improvement and persistent hemiballismus at two-week follow-up reflect this variability and highlight the importance of close outpatient neurological assessment and treatment adjustments.

## Conclusions

This case highlights the clinical complexity and neuroanatomical diversity underlying post-stroke hemiballismus, demonstrating that lesions beyond the classical subthalamic nucleus, such as in the thalamus, can produce similar hyperkinetic movements. The occurrence of hemiballismus following a thalamic hemorrhagic stroke, as presented here, is relatively rare and adds to the limited case-based literature on this presentation. Vascular comorbidities, including hypertension, diabetes, and prior stroke, contribute to both stroke risk and movement disorder manifestation, complicating management. Given the variability in symptom onset and recovery and the absence of standardized treatment protocols, individualized symptomatic therapy and close neurological follow-up are essential. While many cases resolve spontaneously within six months, persistent symptoms require ongoing assessment and tailored management. Further research focused on elucidating pathophysiological mechanisms and optimizing therapeutic strategies is needed to improve outcomes for patients with post-stroke hyperkinetic movement disorders.
